# Foraging and the importance of knowledge in Pemba, Tanzania: implications for childhood evolution

**DOI:** 10.1098/rspb.2023.1505

**Published:** 2023-11-15

**Authors:** Ilaria Pretelli, Monique Borgerhoff Mulder, Bakar Makame Khamis, Richard McElreath

**Affiliations:** ^1^ Max Planck Institute for Evolutionary Anthropology, Leipzig, Germany; ^2^ Institute for Advanced Study in Toulouse, Toulouse, France; ^3^ Department of Anthropology, University of California, Davis, CA, USA

**Keywords:** childhood evolution, foraging, life history, ecological knowledge, Pemba island, Bayesian models

## Abstract

Childhood is a period of life unique to humans. Childhood may have evolved through the need to acquire knowledge and subsistence skills. In an effort to understand the functional significance of childhood, previous research examined increases with age in returns to foraging across food resources. Such increases could be due to changes in knowledge, or other factors such as body size or strength. Here, we attempt to unpack these age-related changes. First, we estimate age-specific foraging returns for two resources. We then develop nonlinear structural equation models to evaluate the relative importance of ecological knowledge, grip strength and height in a population of part-time children foragers on Pemba island, Tanzania. We use anthropometric measures (height, strength, *n* = 250), estimates of ecological knowledge (*n* = 93) and behavioural observations for 63 individuals across 370 foraging trips. We find slower increases in foraging returns with age for trap hunting than for shellfish collection. We do not detect any effect of individual knowledge on foraging returns, potentially linked to information sharing within foraging parties. Producing accurate estimates of the distinct contribution of specific traits to an individual’s foraging performance constitutes a key step in evaluating different hypotheses for the emergence of childhood.

## 1. Introduction

Humans have special life histories, i.e. how individuals trade-off growth, maintenance and reproduction along the life course [1–3]. Across the animal kingdom, larger bodies are often associated with longer lifespans and later reproduction, under the assumption that it pays off to invest in growth for longer periods as long as enough benefits are provided by bigger size during adult life [4]. For example, in many non-human animals, larger bodies are associated with higher fertility and mortality reduction, a main driver of life-history evolution [5]. But other traits can be acquired during development and result in low mortality and high fitness, and thus they have the potential to influence life-history traits. In particular, encephalization, i.e. investment in brain growth, is thought to be at the basis of the slow down of primate and human life histories [6]. But how would encephalization benefit humans? Kaplan *et al.* [7] suggested that several human life-history traits coevolved with our reliance on foraging in complex ecological niches. In what they call Embodied Capital Theory (ECT), they propose that during a long pre-reproductive period, young humans acquire somatic and cognitive traits (the embodied capital) that allow them to extract from the environment high-caloric resources that require high levels of skill. Once adults, humans can then reduce mortality risk and provide for larger families with high-quality food, with a particular emphasis on large game. Alternative hypotheses explain the evolution of long childhoods and slow lives as a consequence of a general elongation of lifespan, where the selective pressure is on survival to older ages [8], the need to develop social cognition [9], or point to inclusive fitness benefits linked to cooperation and resource sharing within human reproductive nuclei [10].

Predictions from these alternative hypotheses have been addressed in numerous ways. For example, González-Forero *et al.* [11], in their simulation of the *Homo* lineage evolution, found an association between encephalization and environmental complexity when modelling the cost of brain tissue as a function of environmental and social challenges. Similarly, a cross-species analysis of niche complexity and life history found that reliance on foods that require high levels of cognitive skills predict slow development in primates [12]. Other types of analysis test hypotheses for the evolution of human life-history relying on data on contemporary hunter–gatherer societies, by tackling one or more predictions associated with the embodied capital model. Several researchers looked at how foraging proficiency varies with age, under the ECT-driven assumption that learning to forage imposes a trade-off between current and future production (i.e. learning diminishes an individual's productivity in the short term but increases it later). Late age at peak foraging, consistent with ECT, has been found in various studies focusing on hunting (e.g. [13–15]), including in a large cross-cultural comparison [16]. Other studies, on the contrary, find early development of foraging skills in other resources, especially fruits and shellfish [17,18], which has been interpreted as contradicting the current versus future production trade-off assumption. But foraging skill acquisition can vary with complexity of the task, so that findings of no strong age effects (e.g. [19]) may be specific to easy tasks. Indeed, Pretelli *et al.* [20] found that, across cultures, more complex resources require more skill and longer acquisition times.

Researchers also tackle the difference between types of ‘embodied capital’. For example, Bock [21] suggests that the need for both somatic and cognitive embodied capital establishes a ratchet mechanism whereby foraging itself promotes the acquisition of the embodied capital, which in turn improve individuals’ ability to forage, a system he defines the ‘punctuated development model’. In support of the necessity for a combination of cognitive and somatic skills for the development of foraging skills, Bock [22] found that arm strength is a predictor of return rates in canoe fishing for boys on the Okavango Delta, in Botswana, while it is not for girls’ basket fishing returns rates. Similarly, Bird & Bliege Bird [23] found that height, as a proxy of stride length, is an important predictor of foraging returns, suggesting that somatic characteristics are an important limiting factor for foraging proficiency among Western Australian Mardu children. Bird & Bliege Bird [23] interpret their finding as inconsistent with ECT, although they provide no parallel test of the impact of different cognitive skills. One major limitation of these otherwise innovative studies, though, is that neither of them provides a measure of the cognitive aspects of embodied capital and uses age as a proxy instead. This is problematic because other time-varying traits covary with age, and parsing out variability in these traits at the individual level is fundamental to define their effect.

Here, we address the challenges of testing the embodied capital model using novel data from a part-time foraging population on the island of Pemba, Zanzibar, Tanzania. We also develop a Bayesian analysis that uses structural equation models to describe nonlinear effects of age and other individual-level traits while accounting for uncertainty at all levels. We first characterize age-specific foraging returns, focusing on the comparison across two different types of resources varying in complexity. Second, we measure the relative contribution to foraging returns of one cognitive trait, i.e. ecological knowledge, and two somatic traits, i.e. height and strength, operationalized as grip strength. Our modelling strategy, which uses simultaneous equations to jointly model individual traits and foraging returns, allows us to compare, for the first time, the importance of the two types of embodied capital across the examined resources using individual-level measures for all traits. Our findings suggest only weak support for the ECT. Accordingly, they prompt a more focused discussion of both the biases that emerge in observational data, and the range of skills that might be acquired during childhood, particularly in contexts where resources are foraged communally.

## 2. Material and methods

### (a) Research location

**Figure 1.**
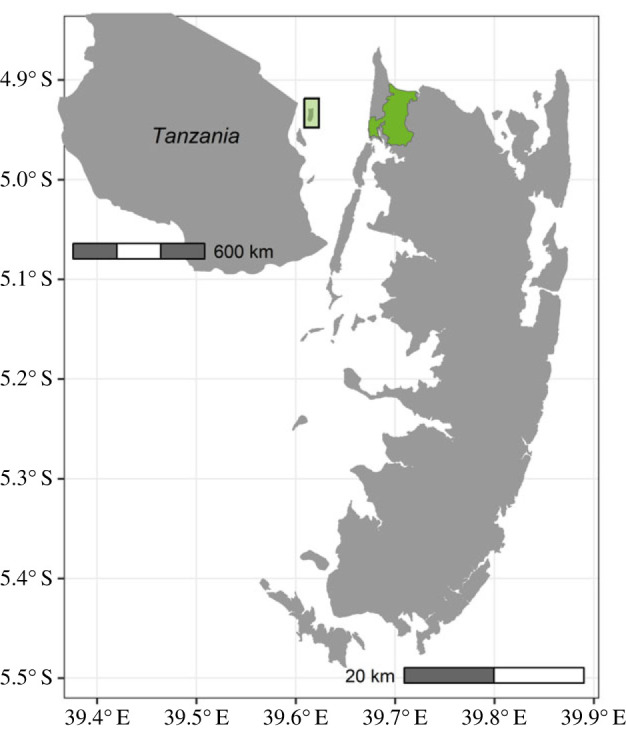
Pemba is an island offshore Tanzania, Ngezi forest (shown in green) is located in its north-western corner.

Data collection was carried out on the island of Pemba, Tanzania. The island is part of the Zanzibar archipelago and is located in the Indian Ocean, about 50 km off the coast of East Africa (figure 1). Pemba’s climate is tropical, with two wetter and two drier seasons brought by alternating monsoonal winds.

Bantu-speaking people have been living on the island since at least 600 AD [24]. Around the turn of the millennium, its inhabitants lived in wattle-and-daub villages and ‘stone-towns’, with coral-rag mosques and multi-storey houses [25]. They cultivated rice, coconuts and cotton [26], and were engaged in long-distance maritime trade that encompassed the whole Indian Ocean [27]. At the end of the fifteenth century, the Portuguese crown took control of the island, only to lose it to the Omani sultanate at the end of the seventeenth century [28]. Although under increasing control from the British, up to the establishment of a protectorate in 1890, the Busaidi sultans remained the formal rulers of the Zanzibar archipelago until 1964, when a revolutionary movement removed them from power and promoted the unification of Zanzibar to mainland Tanganyika, thus forming modern Tanzania.

Pemba is known in Arab texts as al-Jazra al-khadr, or the Green Island, because of its thick forest cover and fertile soils. The primary forest has now been largely replaced by crops, including clove trees, which are the main cash crop on Pemba. The island has few larger wild fauna, but there are several endemic species of birds, bats and other smaller animals. Many of these live in the forest of Ngezi, the largest patch of rainforest that still stands in the north-western corner of the island. The village where data were collected is located in this area, between Ngezi forest and the coast.

### (b) Subsistence and foraging around Ngezi forest

People in the village subsist mainly on agriculture, fishing and the products of the forest, which respectively represent 40.6%, 9.7% and 33% of total income for the average family in this ecozone [29]. Hunting and gathering are a secondary source of food and are carried out primarily by children and young adults, although the foods thus produced regain importance in periods of famines [30]. The present study focuses on two main forms of foraging (which are the most common forms of foraging in the community and thus those for which data collection was the most successful): shellfish collection along the mangrove-fringed coastline and snare hunting in the forest or in the cultivated areas (*shambas*) surrounding the village (which accounted for 80% of all observed foraging trips).

Shellfish are collected by girls of various ages, sometimes including adult women and often accompanied by younger boys—sons or younger brothers. At low tide, groups of foragers walk on the exposed sandy bottom searching and collecting different kinds of shellfish, crabs and occasionally other animals such as small octopuses. Shellfish need to be located, which requires good eyes and experience, then extracted from the sandy bottom, often with the help of a knife and strength. Crabs are often also embedded in the sand, but escape when extracted, so that agility is necessary to capture them. Shellfish collection is not extremely complex, in the sense that the motor skills required are relatively simple, if they do require strength and speed, so that with relatively little experience it is possible to extract the shells from the sand.

**Figure 2.**
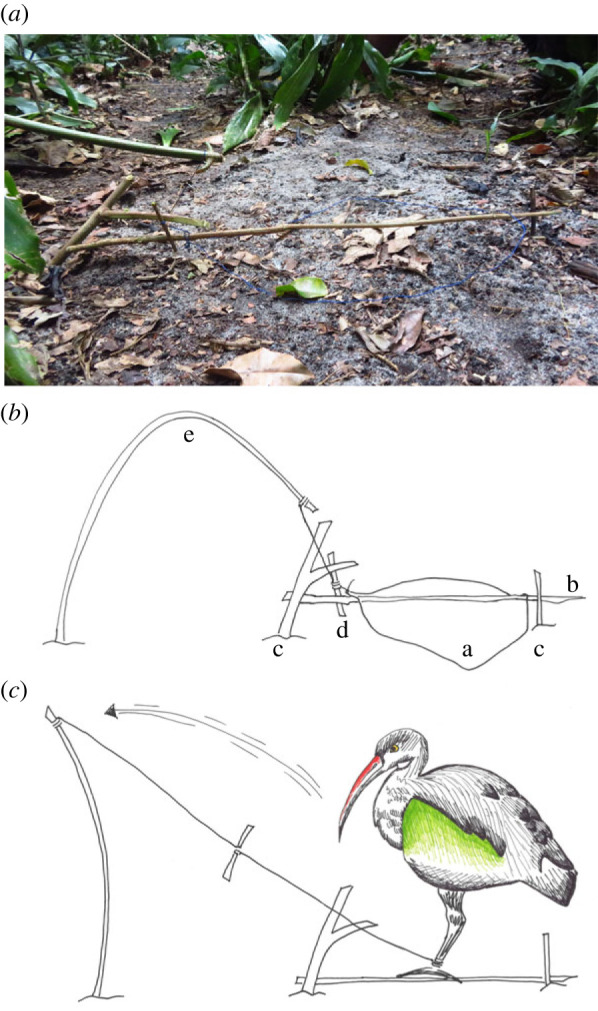
Traps are placed on the forest floor, along paths or where there are signs of animal passage—*a*. The prey is captured by a thread tied into a noose with a slipknot (a, panel *b*). The loop of the noose is placed on top of a stick that acts as a trigger (b). This is kept suspended above the ground by a mechanism composed of two vertical sticks infixed into the ground (c) and another small stick (d) to which the thread of the noose is connected. This thread is also tied to a flexible branch (e), infixed into the ground and kept in a bent position by the rest of the trap mechanism. If a prey steps on the suspended stick (b), as shown in *c*, this activates the trap, so that the flexible branch (e) springs free, tightening the noose around the leg of the prey.

Hunting is almost exclusively practiced by groups of boys, mostly before reproductive age, and involves the use of snares, slingshots, baited traps or gluey sticks targeting birds of various sizes and small mammals, as well as dogs to pursue monkeys or other animals that pose a threat to crops. Young hunters in Pemba are also known to hunt bats, especially the endemic Pemban Flying Fox, *Pteropus voeltzkowi* [31]. In this paper, we focus on trapping with snares. Snares are built using wooden sticks and common sewing threads on the forest floor (figure 2). They are placed along wildlife paths within the forest proper, often close to marshes, or in the casuarine forest closer to the sea, with the aim of capturing ground-dwelling birds including Hadada Ibis (*Bostrychia hagedash*) and the Water Thick-knee (*Burhinus vermiculatus*), or small mammals such as the endemic Pemban Blue Duiker (*Philantomba monticola sundevalli* or *pembae*). Hunting with snares requires understanding the ecology of prey species and tracking skills, in order to appropriately place the traps, as well as dexterity to mount the traps, plus a baseline of strength to bend the long elastic stick that provides momentum to tighten the loop of the snare (see electronic supplementary material, S1, for more details on these subsistence activities). We expect trap hunting to be more complex than shellfish collection, in the sense that it requires a higher degree of coordination to install the trap and knowledge to decide on the position and direction of the trap.

### (c) Data collection

*Demographic data:* with government approval for research (IMMZ/07/17/25, ethics approval provided by Max Planck Institute Ethics Council, application number 2019_05), I.P. visited all households in the village in June 2019. An overview of the project was presented, in order to obtain informed consent, and basic demographic and household-level data were collected for each family unit. A total of 94 households were surveyed and the village census counted 576 individuals in 2019.

*Ecological knowledge data:* a survey instrument was developed during focus groups, in collaboration with adult members of the village and with the Department of Forestry and Non-Renewable Natural Resources in Pemba. Between July and August 2019, I.P. used the survey thus developed to interview 93 individuals aged 5–26, plus a 56-year-old man. Ecological knowledge was measured from a combination of three parts of the survey: answers to a freelist question, where individuals were asked to name all living creatures that could be encountered in and around the village, forest, or beach and sea; answers to 50 ecological knowledge questions; identification of organisms shown in 27 pictures. For more information on this method, see Pretelli *et al.* [32].

*Anthropometric data:* anthropometric data, namely height and hand grip strength, were collected with a stadiometer and hand-held dynamometer, respectively. Height is considered a proxy for body size, stride length and walking speed, which can all influence foraging in the ability and speed when moving in either the forest or the interdidal zone [33]. Hand-grip strength is here used as a proxy for upper body strength, which is necessary both to extract shellfish and to install traps [34]. These measures were collected between one and four times from individuals offering themselves for measurement, including during demographic interviews, knowledge interviews and other impromptu situations. We considered the mean of these measures for each individual. Height was measured for 284 individuals and grip strength for 258 individuals.

*Behavioural observations:* between February and December 2020, children planning a foraging trip were invited to inform I.P. or B.M.K. so that behavioural observations could be carried out. A value of 1000 TSH (about the equivalent of 0.40$) was paid to the group to promote the communication of upcoming trips—an amount sufficient to buy two small packets of biscuits or a couple of pencils, but also enough to ensure that the researchers could observe foraging trips. For each trip, we recorded the following information: group composition, start and end time from a location in the village, time of arrival and departure from destination of the trip (i.e. the shore where shellfish are collected, or the area in the forest where traps are mounted), and GPS track of the movement of the group.

Moreover, each participant in shellfish collection trips was provided with a small bucket of which the contents were weighed at the end of the trip in order to measure individual-level returns. Time and height of low tide was recorded on the day of each foraging trip from the website Tide Charts (https://www.tideschart.com/Tanzania/Pemba-North/Micheweni/Konde/). In total, 39 individuals participated in 35 shellfish collection trips, which gave a total of 156 person-trips. Of these, 72% are relative to female foragers, and 8–39 is the age range for person-trips data (mean 16.1 years).

Additional trap-level information was recorded during foraging trips that involved installing and checking traps in the forest, including who installed each trap, when a trap was installed, checked and dismantled, whether a trap captured something and, if so, the weight of the prey. In total, 53 individuals participated in snare hunts and, of these, 24 individuals installed a total of 724 snares during 335 foraging trips. The outcome measure considered in the analysis was the number of times each trap captured a prey. In total, 31 traps captured anything and only two of these captured a prey more than once (see data in figure 4*b*). All observed hunters were males, aged 7–26, and the mean age of the individuals installing traps was 15.2 years. Information on sample sizes is provided in table 1.

**Table 1.** Sample size of the data used in the study.

type of foraging	N. participants	N. trips	N. outcomes	definition of outcome
shellfish collection	39	35	156	kg of unprocessed shellfish collected by one participant during one trip
trap hunting	24	335	724	number of prey captured by a trap mounted by one individual

### (d) Causal framework

**Figure 3.**
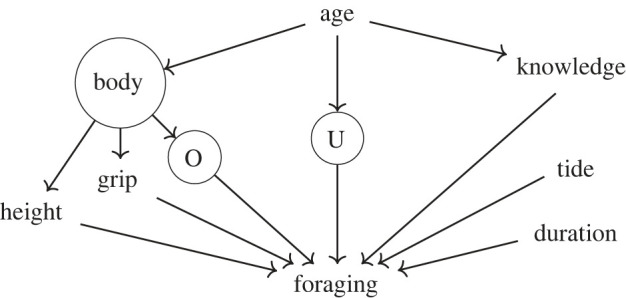
DAG describing relationships between analysed variables. Note that U marks unknown age-varying factors that affect foraging returns, while O stands for somatic traits other than height or grip strength that have not been measured.

The directed acyclic graph in figure 3 shows the factors that are believed to influence foraging. The first step in our analysis is a description of how foraging varies with age, for which we look at the total effect of age and include only age as a predictor of foraging at the individual level. But age itself does not ‘cause’ foraging, rather its total effect is mediated by time-varying covariates, such as knowledge or somatic characteristics. In addition to these measurable traits, several other unmeasured or unmeasurable time-varying traits, including, for example, ability with a knife or patience, influence foraging, and are designated in the DAG with the letter U. For the second step in our analysis, we take into consideration the separate effects of ecological knowledge, as measured by our survey instrument, and anthropometric measures, namely height and grip strength, which stand as proxies, respectively, for body size (e.g. length of legs and stride length) and somatic strength. Additional somatic traits that have not been measured might influence foraging returns; hence, we include in this graph an additional unknown variable O. Note that the effect of these other unmeasured traits is likely partially captured by age, strength or height. Both somatic and cognitive traits are correlated with age, and thus with each other, but controlling for age in this model makes them conditionally independent. In this way, we can estimate their effect within the same statistical model. Moreover, we include in all models trip-level covariates such as duration and, in the case of shellfish collection, tide height. Note that these variables are not confounders on the path of age, but are concurrent causes of foraging returns and could introduce biases if not controlled for (for additional considerations on causal connections between individual-level traits, such as knowledge, and trip-level traits, see electronic supplementary material, S2). Finally, with the exception of tides, this causal structure holds for both types of foraging considered here, even though the factors might act in different ways. In this paper, we address two causal queries separately, using two different sets of model. First, we describe the variation with age in foraging performance, defined as either success of traps or amount of shellfish collected. For this goal, we use a statistical model that includes only age as an individual-level predictor. In a second stage, we include individuals’ height, grip strength and knowledge as predictors of foraging performance in order to understand their relative importance.

### (e) Analysis

Foraging returns are most appropriately modelled as hurdle models, modelling separately (i) the probability of success (i.e. non-zero returns) and (ii) the amount of returns (e.g. grams or calories collected) for a certain trip [14,16,20,35]. In the case of the present study, however, the two types of foraging are analysed with just one of the two parts of a hurdle model. This is because, for hunting with traps, we mainly model the probability that each trap has of collecting anything at all, i.e. the first part of a hurdle model, given that the weight of the captured animal is most likely independent of the modelled individual traits (weight of prey depends mainly on the species captured, which varies seasonally, see electronic supplementary material, figure S1). For collecting seashells, the second part of the hurdle model is sufficient, because children always obtain something–hence no need to model zero returns separately.

Mathematically, the amount of shells collected by children is a lognormally distributed quantity, where the mean of the underlying normal distribution is *μ* and the standard deviation is *σ*. The number of times a trap captured an edible prey is a Poisson distributed outcome with rate *λ*.

2.1 R∼lognormal(μ,σ)

and

2.2 S∼Poisson(λ).

The parameters *μ* and *λ* are modelled in parallel, so that the effect of age and other individual level traits can be compared. Appropriately transformed, both are a combination of a general intercept, *α*, an equation including the effects of individual-level traits, designed by *ϕ*, and an equation for trip-level traits, designed by *ψ*.

2.3 $$\mu =\log(\alpha \;\phi_{i}\;\psi_{f})$$

and

2.4 $$\lambda =\exp(\alpha \;\phi_{i}\;\psi_{f}).$$

The first parameter in the equations defining *λ* and *μ* is *α*, which simply scales all other parameters and allows them to fit to the data.

*ϕ*_*i*_ is calculated for each individual *i* and includes individual-level random effects, $\iota_{i}$, and a series of factors that are tied together in a production function akin to a Cobb–Douglas (see electronic supplementary material, section S3, for more details). In particular, age *a* is scaled by parameters *β* and *γ* so that its effect on foraging can grow in a flexible, sigmoidal function before levelling off at 1 (see electronic supplementary material, figure S7). Knowledge *k*, height *h* and grip strength *g* are included only in the second set of our models (see electronic supplementary material, S2.4) and are scaled by exponents *ζ*, *η* and *θ*, respectively, as it is the norm in Cobb–Douglas functions. This structure allows us to incorporate some basic biological assumptions in the model: for example, in the absence of any knowledge, height or grip strength, foraging cannot happen. For more details, refer to electronic supplementary material, S3.

2.5 $$\phi_{i}=\iota_{i}\;{\left(1-\exp(-\beta \;a_{i})\right)}^{\gamma }\;k_{i}{\;}^{\zeta }\;h_{i}{\;}^{\eta }\;g_{i}^{\theta }.$$

Moving on to *ψ*_*f*_, this parameter includes all information relative to *f*, i.e. the foraging trip, for shellfish or trap, for the snare data. These include a time-scaling parameter, *ξ* which moderates the effect of the duration *d* of a foraging trip in minutes, for shellfish, or of duration of exposure, i.e. number of days a trap was deployed in the forest, for trap hunting. Collecting shellfish is also influenced by the depth of the tide *t*, so that *ψ* for this kind of data includes the effect of average height of the tide during the foraging trip, multiplied by *τ*.

2.6 $$\psi_{f}=d_{f}^{\xi }\;\exp(t_{f}\tau ).$$

Other aspects of the models include the estimation of ecological knowledge using an Item Response Theory model (see Pretelli *et al.* [32] for more details on this tool) and the estimation of missing data for knowledge, height and grip strength according to each individual’s age and sex. Note that even though data points relative to individuals who are missing certain measurements provide no information to the model concerning the specific inference for knowledge, height or grip strength, including these data points still improve the model fit and estimation of the other parameters, informed by the non-missing parts of the data (see electronic supplementary material, S3.2).

We used weakly informative or informative priors for all parameters, reported here for the parameters listed above (see electronic supplementary material, S3.1 for the remaining parameters and for a visual description of the joint distribution of the prior in electronic supplementary material, figure S14).

$$\begin{array}{rl} & \alpha ∼\text{half-normal}(0,1),\\ & \beta ,\gamma ,\sigma ∼\text{exponential}(1)\\ \mathrm{and}\; & \iota ,\zeta ,\eta ,\theta ,\xi ,\tau ∼\text{normal}(0,1). \end{array}$$

Hamiltonian Monte Carlo engine Stan [36] was used to estimate the posterior distribution for each parameter using CmdStan. Posterior distributions were processed in R v. 4.3.1. [37], with the help of the Statistical Rethinking R package, v. 2.31 [38]. Our models were validated with simulated data to ensure that they could recover the simulated parameters (see electronic supplementary material, S3.3) and, for all analyses, visual inspection of the trace plots, Gelman–Rubin diagnostic and the effective number of samples indicate model convergence.

Parameter estimation in the Bayesian framework does not return point estimates, but rather a posterior distribution of possible values for a parameter. Hence, all the figures in the results section convey information relative to the whole posterior distribution, when possible. Moreover, rather than as effect sizes, our results are reported as counterfactuals, i.e. the expected outcomes the model predicts under certain conditions. For more information, refer to electronic supplementary material, S3.4. Code and data necessary to reproduce the results presented in the main text are available on Dryad.com under the doi:10.5061/dryad.c866t1gcr.

## 3. Results

**Figure 4.**
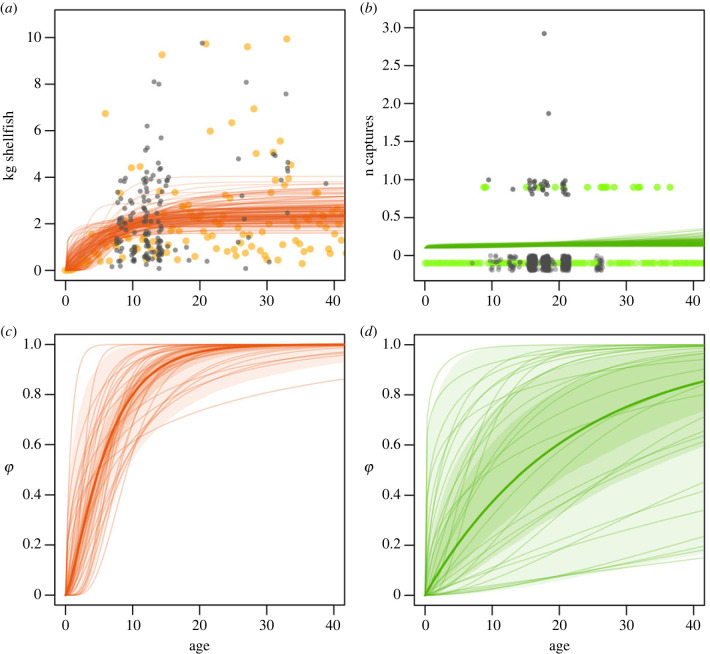
Panels *a* and *b* show, for shellsh collection and trap hunting, respectively, (i) raw data (grey dots), i.e. kg of unprocessed shellfish or number of successes of traps, as a function of age of participant; (ii) curves showing 150 posterior predictions for expected outcome values, by age, as predicted by the models, i.e. average amount of shellfish an individual of a certain age is expected to collect in a fixed amount of time–orange lines in panel *a*, or the probability that a trap set by a boy of a certain age has of capturing a certain number of prey during an average exposure time–green lines, panel *b*; (iii) data points generated by the posterior predictions (coloured dots), i.e. simulated kg of shells per trip or successes of traps. Note that points illustrating zero success for both the raw and simulated data in panel *b* are placed below zero on the *y*-axis and all points are jittered for clearer visualization. Panels *c* and *d* show the age component of this variation, stripped of the effect of other predictors, i.e. the average value of *ϕ* for each age. Considering *ϕ*, which is a dimensionless variable defining the total effect of individual characteristics, allows comparison between the two types of foraging, despite the different scales at which the actual outcomes are measured. Shaded areas in panels *c* and *d* show the 30th, 60th and 89th percentiles, and the overlying curves show 30 samples drawn from the posterior distribution of parameters. The results presented in this figure are given by a model that includes only individual-level random effects and an effect of age to define *ϕ*–refer to equation (2.5), but without predictors for knowledge, height or grip strength.

*Fit to data.* Before discussing the inferences from our results, we must point out some differences between the types of data we analyse. The grey points in figure 4*a*,*b*, show our raw data, i.e. kg of unprocessed shellfish or number of successes of trap, respectively, as a function of the age of the participant. A first visual inspection of these data reveals that, despite having received substantially more sampling effort (335 foraging trips observed over 11 months, with a total of 985 person/trip), trap hunting yielded relatively low-definition data: only 24 participants actually built traps during these trips, the age range does not cover adult individuals, with the oldest man in the sample only 26, and traps have overall a small success rate (only 31 out of 724 traps captured something edible). By contrast, the dataset for shellfish collection is relatively well defined from childhood to early adulthood, with sparser but still helpful data up to mid-adulthood. This difference has consequences for the precision with which we can make inferences relative to the two types of foraging: shellfish data provide clearer and more reliable estimates than trap hunting across all of our results. For example, we can see this by the higher precision with which our models define age trajectories for shellfish data in figure 4*a*,*c*, compared with the less confident estimates relative to trap hunting, in *d* (refer to electronic supplementary material, figure S13 to observe the uncertainty in the equivalent of panel *a*). Model validation shows that a sample size of 500 trips performed by 30 individuals would be sufficient to recover clear parameter estimates as long as the success rate is sufficiently high. The current uncertainty is thus likely a feature of the low success rate in our sample (see electronic supplementary material, S3.3). Despite this, both models successfully predict outcomes that match the observed data, as we can infer by the extent by which the predicted outcomes–coloured points–match the observed data—grey points—in figure 4*a*,*b* (see also electronic supplementary material, S3). So, keeping in mind that results are less confident for trap hunting, we can look at central tendencies in the posterior distributions and proceed to describe our results.

*Variation with age.* Our first target of inference is how foraging returns varies with age, for which the two types of foraging we analyse show very different patterns (figure 4*c*,*d*). While, on average, a 10-year-old individual collecting shellfish has reached 80% of the maximum foraging returns, a 10-year-old boy who goes hunting with traps has achieved only about 30% of the maximum success rate individuals can be expected to achieve. In general, foraging returns increase at a much faster rate for shellfish collection than for trap hunting.

**Figure 5.**
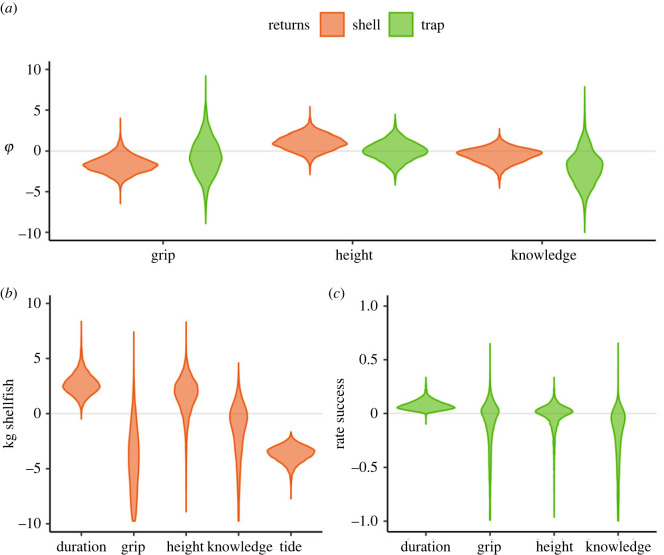
Effects of individual- and trip-level traits on foraging returns, where positive values mean that higher values of the trait correspond to higher values at the outcome level, and *vice versa*. For example, in *b*, longer duration of the trip implies more returns, as shown by the distribution largely above zero for duration, while lower tide levels mean higher foraging returns, as shown by the very negative distribution for tide. Panel *a* compares the effect of grip, height and knowledge on returns for shellfish and trap returns. These are shown as counterfactual contrasts for the latent parameter *ϕ*. In the statistical model, *ϕ* summarizes the total variation due to individual differences on foraging returns, including the effect of the somatic and cognitive traits of that individual. By calculating counterfactual values, i.e. values of *ϕ* for hypothetical individuals who have average values in all traits but one, extremely high, or low value for the remaining trait (e.g. average height and strength, but exceptional knowledge), we can visualize how important each trait is for foraging returns. By focusing on *ϕ*, we can compare the effect of each trait on shellfish versus trap foraging returns, because *ϕ* is an adimensional parameter scaled similarly across the two resources. Panels *b* and *c* show the effect of each of the main predictors in the sample (excluding age) at the outcome level, i.e. kg of shellfish or rate of success of traps. This is the difference in average amount of shellfish produced in two counterfactual conditions where all variables are kept at mean value apart from the one under evaluation, for which maximum and minimum values are contrasted. See electronic supplementary material, S3.4 for more information on how counterfactual values are calculated and electronic supplementary material, figure S14, to see how these counterfactual contrasts compare with the priors provided to the model.

*Knowledge, strength, height.* As a second step, we aim at partitioning the relative contribution of cognitive versus somatic traits for foraging. The former are represented by ecological knowledge, and the latter by height and grip strength. We find a positive effect of height on foraging for shellfish collection (figure 5*a*), a negative effect of grip strength on shellfish collection, and a slightly negative effect of ecological knowledge on trap hunting and on shellfish collection. Finally, the effect of grip strength and height on trap hunting is unclear, given that the model is mostly recovering the priors we imposed on the model, although if any, their effect is likely to be limited (see electronic supplementary material, figure S14).

*Other predictors: time and tide.* In addition to individual-level traits, we considered trip-level traits for shellfish collection, that is, duration of the foraging trip and average level of the tide during the trip, and duration of exposure of a trap for the trap-hunting data, that is, number of days a trap was deployed. These trip-level factors appear to be the most important predictors for shellfish collection data. Longer trips yield significantly more shellfish, as each unit of time increases total shellfish production. Moreover, there is a strong positive effect of the average water level on returns, with higher returns associated with a more extreme low tide. Finally, duration of exposure has a positive effect on the number of prey captured by a trap.

## 4. Discussion

In this study, we aimed to measure the relative contribution of cognitive and somatic traits for foraging returns. This is important for assessing the significance of hypotheses for the evolution of human childhood. Our results replicate some of the findings from previous research at other sites over the last three decades, specifically low success rate and late development of skill for hunting (e.g. [13,15,16]), earlier acquisition of easier skills such as shellfish collection [20,39] and positive effect of body size on such collection [39]. To do so, we collected novel data, including behavioural observations, for two different types of foraging, together with anthropometric and demographic measures, and also, for the first time, an explicit measure of ecological knowledge. We built on existing literature (e.g. [16,20]) to develop a statistical model based on explicit causal assumptions, which helps us tackle more precisely the question of why humans spend so long as pre-reproductive individuals.

Compared with previous studies, we improve the statistical methodology by not only including a principled treatment of missing data, but also by analysing children’s ecological knowledge using IRT, whereby we can maximize the information yielded from different tasks (questions) across a protocol that is necessarily quite difficult to construct. We also provide a structural equation model approach to estimating impacts on productive activity on both individual- and trip-level traits, which can contribute to better controlled investigations into individual-level effects in the future.

Our first result, in agreement with previous studies, is an early-age increase of foraging returns for shellfish collection, and, on the contrary, late development for trap hunting. This is overall in accordance with ECT, as foragers take longer to become proficient hunters, an activity that is deemed to require a more complex set of skills.

However, we do not find that ecological knowledge positively affects shellfish collecting or trapping returns. This is one of the predictions of ECT, as individuals who spent longer time learning about the environment are expected to have higher foraging returns. Several limitations inherent to our dataset and the specific ethnographic context could account for this finding. For example, we do not include foraging types such as big game hunting, which is considered to require both ecological knowledge and skill. In addition, the types of foraging we target are only two of the many in which Pemban children engage, while our measure of individual-level knowledge is non-specific and includes information relative to medicinal plants, pelagic fish, pests, etc. (see electronic supplementary material, S1.4). Moreover, our data are observational and, as such, do not randomize knowledge of individual foragers, potentially introducing biases. For example, we may be observing only the most knowledgeable individuals foraging, so that we cannot pick up a positive effect of having learned about the environment.

Still, it is important to note that all activities considered are performed socially. With a median group size of three and four foragers, respectively, both trap hunting and shellfish collection are almost always carried out in groups. This means that whatever knowledge is necessary for these activities, this can often be shared within the group as long as at least one individual possesses it. For example, when boys go on a trip to build traps, they generally take responsibility for building one trap each, but also keep sharing information and offer counselling. Moreover, decisions are taken communally on the area in the forest where to build a set of traps. Trap building, then, becomes a discussion between individuals building several traps at the same time, coordinating and suggesting to move the location of the trap or to use a different stick. For shellfish collection, we might be observing something that has been suggested for the harvest of fruits as well [20]: knowledge is necessary during these activities in order to locate high-yield patches and, maybe more importantly, to know when these seasonal resources will be available. In the case of shellfish collection, additional temporal variability is given by the tidal cycles. Tide level is important for shellfish collection because it influences how many resources are available. Even though this activity is always carried out at low tide, there is considerable variation in how much the water retreats, as minimum yearly tides are observed only in the presence of certain astronomical conditions. This has implications for shellfish gathering, not only because the lower the tide, the larger the exposed area of sandy bottom, but also because the areas that are exposed less often are also less exploited and likely to yield higher returns. Hence, given that tides are the best predictor for foraging returns, it is important to know when a tidal minimum is going to happen. But, as long as one person per group has this information, everyone else will enjoy the benefits of foraging at low tide. This means that, although ecological knowledge of foraging individuals does not appear to promote higher individual-level returns, the knowledge available to foragers and shared within the group can nevertheless be relevant for the foraging of all participants.

Concerning other types of ‘embodied capital’, one interesting result is the positive effect of height on shellfish collection. This replicates previous findings of Bird & Bliege Bird [39], who argue that, in accordance with predictions of optimal foraging theory [40], encounter rate is one of the main determinants of shellfish collection efficiency. When foraging for sessile organisms, the rate of encounter is related to the speed of the forager, which, for humans, is usually correlated with height and stride length. Optimal foraging theory also predicts a shift in the prey-set foragers target as the encounter rate with favoured prey changes. In our data, for example, we observe a smaller size of adult’s prey-set, which only include two or three preferred types of shellfish, while children and teenagers collect up to 8 or 10 different types (see electronic supplementary material, figure S3). Note that adults might be targeting species with better shell/meat ratios so that, while the total weight of unprocessed catch does not change much at later ages, the caloric content of the catch might increase. Finally, for both grip strength and height, the data are not sufficient to reveal a clear pattern in the case of trap hunting.

One more aspect to consider is the underrepresentation of adults in our sample. This is, in part, a feature of our data collection procedures, which explicitly targeted younger individuals. But ethnographic understanding supports the idea that adults do not forage nearly as much as children and teenagers do. In particular, for trap hunting, we are aware of only one adult man who occasionally practices the activity. This might be due to the fact that hunting in the forest is restricted by law and adults might either refrain from hunting because of legislation or might fail to report it to researchers (see electronic supplementary material, S1). But hunting with traps has very low success rates, and relatively high costs in terms of time. Simply put, most adult men might have better things to do. Shellfish collection, on the contrary, can be a reasonably profitable activity: when the tide is low, the average forager is expected to collect more than 3 kg of unprocessed shellfish in a 3 h long trip. Still, adults are underrepresented in our sample. This might be due to the fact that adults, according to our models, do not have higher return rates than teenagers, and they might prefer to delegate collecting shellfish to their children, when possible, given the starker opportunity costs adults face when foraging instead of performing other activities.

Overall, although at least some of our results do not explicitly support ECT, they are also consistent with several other non-exclusive possibilities: long pre-reproductive periods might be driven by skills for the most difficult resources only, or those where the skills of all foragers are relevant, e.g. communal hunting. Selection could also act through composition of the foraging parties and specialization: if each type of foraging requires at least one highly skilled forager, individuals who take a long time to acquire specialized knowledge contribute to certain types of foraging trips, and also get the benefits of participating in other foraging activities led by other specialized individuals. ECT may also have favoured the emergence of traits such as selective imitation and attentiveness to other individuals’ level of skill, or, more generally, even though social foraging might weaken the strength of selection on ECT-related skill levels, it might promote the emergence of facilities whereby an individual can become part of a group with a skillful forager. In other words, we may still need to broaden further the cognitive aspects of embodied capital from individual skill levels to more diffuse aspects of sociality acquired during childhood.

Our results indicate that childhood is a not time for idling, or just a by-product of other selective pressures, as suggested by early hypotheses [8]. On the contrary, we show that childhood is an active period during which individuals engage, sometimes very successfully, in a variety of activities, which promotes knowledge acquisition [32]. Learning can still be an important reason for the evolution of childhood, however, we point to the fact that the pathways by which knowledge impacts results, and fitness down the line, can be more complex than expected and implicate the involvement of other individuals. Moreover, our results are also consistent with an elongation of the pre-reproductive phase pushed by inclusive fitness benefits, as most of the foraged goods were shared either with the family or the foraging party—which often includes siblings.

In conclusion, we find that complexity of resources influences age trajectories in foraging returns, but also that returns are not improved by individual level ecological knowledge. However, we emphasize again the complexities of the human foraging niche, and the study thereof. Many different elements contribute to the foraging returns in each type of hunting or gathering. These often vary during the lifetime of foragers due to development or ageing, but also due to societal and technological changes, complicating the analysis of longitudinal foraging data, which themselves are fundamental to addressing time-varying questions. Moreover, societies perform multiple types of foraging, and, in order to appropriately address each of them, it is important to have an exhaustive ethnographic understanding of the subsistence strategies and how they vary across the lifetime, socio-economic status etc. Finally, inferences that can be generalized to the whole human species require cross-cultural analyses that additionally complicate the goal of taking into account these ethnographic details. Although more conclusive tests for hypotheses on the evolution of childhood are yet to come, here, we pave the road for future studies by clearly estimating the contribution of individual-level traits to foraging. This will help us understand the processes leading to the evolution of childhood.

## Data Availability

Data used for this paper are available at Dryad: doi:10.5061/dryad.c866t1gcr [41]. Supplementary material is available online [42].

## References

[1] Jones JH. 2011 Primates and the evolution of long, slow life histories. Curr. Biol. **21**, R708-R717. (10.1016/j.cub.2011.08.025) 21959161 PMC3192902

[2] Kaplan HS. 1997 The evolution of the human life course. In *Between Zeus and the Salmon* (eds KW Wachter, CE Finch). Washington, DC: National Academy Press.

[3] Leigh SR. 2002 Evolution of human growth. Evol. Anthropol. **13**, 223-236. (10.1002/evan.20002)

[4] Charnov EL. 1989 Natural selection on age of maturity in shrimp. Evol. Ecol. **3**, 236-239. (10.1007/BF02270724)

[5] Kingsolver JG, Pfennig DW. 2004 Individual-level selection as a cause of Cope’s rule of phyletic size increase. Evolution **58**, 1608-1612. (10.1111/j.0014-3820.2004.tb01740.x) 15341162

[6] Kuzawa CW et al. 2014 Metabolic costs and evolutionary implications of human brain development. Proc. Natl Acad. Sci. USA **111**, 13 010-13 015. (10.1073/pnas.1323099111) PMC4246958 25157149

[7] Kaplan H, Hill K, Lancaster J, Hurtado AM. 2000 A theory of human life history evolution: diet, intelligence, and longevity. Evol. Anthropol.: Issues, News Rev. **9**, 30. (10.1002/1520-6505(2000)9:4<156::AID-EVAN5>3.0.CO;2-7)

[8] Hawkes K, O’Connell JF, Blurton Jones N, Alvarez H, Charnov EL. 1998 Grandmothering, menopause, and the evolution of human life histories. Proc. Natl Acad. Sci. USA **95**, 1336-1339. (10.1073/pnas.95.3.1336) 9448332 PMC18762

[9] Dunbar RIM. 1998 The social brain hypothesis. Evol. Anthropol.: Issues, News Rev. **6**, 178-190. (10.1002/(SICI)1520-6505(1998)6:5<178::AID-EVAN5>3.0.CO;2-8)

[10] Kramer KL, Ellison PT. 2010 Pooled energy budgets: resituating human energy-allocation trade-offs. Evol. Anthropol. **19**, 136-147. (10.1002/evan.20265)

[11] González-Forero M, Faulwasser T, Lehmann L. 2017 A model for brain life history evolution. PLoS Comput. Biol. **13**, 1-28. (10.1371/journal.pcbi.1005380) PMC5344330 28278153

[12] Schuppli C, Graber SM, Isler K, van Schaik CP. 2016 Life history, cognition and the evolution of complex foraging niches. J. Hum. Evol. **92**, 91-100. (10.1016/j.jhevol.2015.11.007) 26989019

[13] Gurven M, Kaplan H, Gutierrez M. 2006 How long does it take to become a proficient hunter? Implications for the evolution of extended development and long life span. J. Hum. Evol. **51**, 454-470. (10.1016/j.jhevol.2006.05.003) 16797055

[14] McElreath R, Koster J. 2014 Using multilevel models to estimate variation in foraging returns: effects of failure rate, harvest size, age, and individual heterogeneity. Hum. Nat. **25**, 100-120. (10.1007/s12110-014-9193-4) 24522975

[15] Walker R, Hill K, Kaplan H, McMillan G. 2002 Age-dependency in hunting ability among the Ache of eastern Paraguay. J. Hum. Evol. **42**, 639-657. (10.1006/jhev.2001.0541) 12069505

[16] Koster J et al. 2020 The life history of human foraging: cross-cultural and individual variation. Sci. Adv. **6**, 1-8. (10.1126/sciadv.aax9070) PMC7314517 32637588

[17] Bird RB, Bird DW. 1995 Children and traditional subsistence on Mer (Murray Island), Torres Strait. Aust. Aborig. Stud. **1**, 2-17.

[18] Crittenden AN, Conklin-Brittain NL, Zes DA, Schoeninger MJ, Marlowe FW. 2013 Juvenile foraging among the Hadza: implications for human life history. Evol. Hum. Behav. **34**, 299-304. (10.1016/j.evolhumbehav.2013.04.004)

[19] Bliege Bird R, Bird DW. 2002 Constraints of knowing or constraints of growing? Hum. Nat. **13**, 239-267. (10.1007/s12110-002-1009-2) 26192759

[20] Pretelli I, Ringen E, Lew-Levy S. 2022 Foraging complexity and the evolution of childhood. Sci. Adv. **8**, eabn9889. (10.1126/sciadv.abn9889) 36223468 PMC9555775

[21] Bock J. 2002 Learning, life history, and productivity: children’s lives in the Okavango Delta, Botswana. Hum. Nat. **13**, 161-197. (10.1007/s12110-002-1007-4) 26192757

[22] Bock J. 2005 What makes a competent adult forager? In *Hunter-Gatherer Childhoods* (eds BS Hewlett, ME Lamb), pp. 109–128. New Brunswick: AldineTransactions.

[23] Bird DW, Bliege Bird R. 2005 Mardu children’s hunting strategies in the Western Desert, Australia: foraging and the evolution of human life histories. In *Hunter gatherer childhoods*, pp. 129–146. New York, NY: AldineTransaction.

[24] Fleisher J, La Violette A. 2013 The early Swahili trade village of Tumbe, Pemba Island, Tanzania, AD 600-950. Antiquity **87**, 1151-1168. (10.1017/S0003598X00049929)

[25] LaViolette A, Fleisher JB. 2018 Developments in rural life on the Eastern African coast, A.D. 700–1500. Taylor Francis **43**, 380-398. (10.1080/00934690.2018.14896619)

[26] Walshaw SC. 2010 Converting to rice: urbanization, Islamization and crops on Pemba Island, Tanzania, AD 700-1500. World Archaeol. **42**, 137-154. (10.1080/00438240903430399)

[27] Fleisher J, Lane P, Laviolette A, Horton M, Pollard E, Quintana Morales E, Vernet T, Christie A, Wynne-Jones S. 2015 When did the Swahili become maritime? Am. Anthropol. **117**, 100-115. (10.1111/aman.12171) 25821235 PMC4368416

[28] Prestholdt J. 2018 Navigating the early modern world Swahili polities and the continental–oceanic interface. In *The Swahili World* (eds A LaViolette, S Wynne-Jones), pp. 517–528. London, UK and New York, NY: Routledge.

[29] Andrews J, Borgerhoff Mulder M. 2022 Forest income and livelihoods on Pemba: a quantitative ethnography. World Dev. **153**, 105817. (10.1016/j.worlddev.2022.105817)

[30] Walsh M. 2009 The use of wild and cultivated plants as famine foods on Pemba Island, Zanzibar. Études Océan Indien **42–43**, 217-241. (10.4000/oceanindien.793)

[31] Walsh MT. 1995 Eating bats on Pemba island. October **6**, 15-18.

[32] Pretelli I, Mulder MB, McElreath R. 2022 Rates of ecological knowledge learning in Pemba, Tanzania: implications for childhood evolution. Evol. Hum. Sci. **4**, e34. (10.1017/ehs.2022.31) 37588933 PMC10426123

[33] Bohannon RW. 1997 Comfortable and maximum walking speed of adults aged 20 to 79 years: reference values and determinants. Age Ageing **26**, 15-19. (10.1093/ageing/26.1.15) 9143432

[34] Bohannon RW. 2019 Grip strength: an indispensable biomarker for older adults. Clin. Interv. Aging **14**, 1681-1691. (10.2147/CIA.S194543) 31631989 PMC6778477

[35] Lew-Levy S, Ringen EJ, Crittenden AN, Mabulla IA, Broesch T, Kline MA. 2021 The life history of learning subsistence skills among Hadza and BaYaka foragers from Tanzania and the Republic of Congo. Hum. Nat. **32**, 16-47. (10.1007/s12110-021-09386-9) 33982236 PMC8208923

[36] Stan Development Team. 2021 Stan Modeling Language Users Guide and Reference Manual. URL https://mc-stan.org.

[37] R Core Team. 2021 R: A Language and Environment for Statistical Computing. URL https://www.r-project.org/.

[38] McElreath R 2020 Statistical rethinking. Boca Raton, FL: CRC Press.

[39] Bird DW, Bliege Bird R. 2002 Children on the reef: slow learning or strategic foraging? Hum. Nat. **13**, 269-297. (10.1007/s12110-002-1010-9) 26192760

[40] Winterhalder B, Smith EA. 2000 Analyzing adaptive strategies: human behavioral ecology at twenty-five. Evol. Anthropol. **9**, 51-72. (10.1002/(SICI)1520-6505(2000)9:2<51::AID-EVAN1>3.0.CO;2-7)

[41] Pretelli I, Borgerhoff Mulder M, Makame Khamis B, McElreath R. 2023 Foraging and the importance of knowledge in Pemba, Tanzania: implications for childhood evolution. Dryad Digital Repository. (10.5061/dryad.c866t1gcr). PMC10646471 37964531

[42] Pretelli I, Borgerhoff Mulder M, Makame Khamis B, McElreath R. 2023 Foraging and the importance of knowledge in Pemba, Tanzania: implications for childhood evolution. Figshare. (10.6084/m9.figshare.c.6915890) PMC10646471 37964531

